# Effect of Topical PTH 1-34 Functionalized to Biogran^®^ in the Process of Alveolar Repair in Rats Submitted to Orchiectomy

**DOI:** 10.3390/ma15010207

**Published:** 2021-12-28

**Authors:** Paula Buzo Frigério, Pedro Henrique Silva Gomes-Ferreira, Fábio Roberto de Souza Batista, Juliana Moura, Idelmo Rangel Garcia Júnior, Daniele Botticelli, Paulo Noronha Lisboa-Filho, Roberta Okamoto

**Affiliations:** 1 Department of Diagnosis and Surgery, Araçatuba Dental School, São Paulo State University Júlio de Mesquita Filho—UNESP, Aracatuba 16015050, SP, Brazil; pedroferreirabmf@gmail.com (P.H.S.G.-F.); fabiorsbatista@gmail.com (F.R.d.S.B.); juliana.moura032@outlook.com (J.M.); idelmo@foa.unesp.br (I.R.G.J.); 2 ARDEC Academy, 47923 Rimini, Italy; daniele.botticelli@gmail.com; 3 Department of Physics, School of Sciences, São Paulo State University Júlio de Mesquita Filho—UNESP, Bauru 17033360, SP, Brazil; paulo.lisboa@unesp.br; 4 Department of Basic Sciences, Araçatuba Dental School, São Paulo State University Júlio de Mesquita Filho—UNESP, Aracatuba 16015050, SP, Brazil; roberta.okamoto@unesp.br

**Keywords:** osteoporosis, orchiectomy, bone regeneration, parathyroid hormone, biomaterial, men

## Abstract

(1) Background: There are many therapies for osteoporosis control and bone maintenance; anabolic drugs such as teriparatide and bone grafts help in the repair process and stimulate bone formation. Thus, the aim of the present study was to evaluate the behavior of repaired bone in the presence of PTH (teriparatide) associated with Biogran^®^ (biomaterial) through a sonochemical procedure after extraction in rats. (2) Methods: The insertion of Biogran^®^ with PTH in the alveolus was performed 30 days after incisor extraction. Euthanasia occurred after 60 days. (3) Results: The use of local treatment of PTH loaded with Biogran® in healthy rats promoted good results for micro-CT, with an increase in percentage and bone volume, number and trabecular separation and less total porosity. Greater immunostaining for Wnt, β-Catenin and osteocalcin proteins and lower expression for Thrombospondin-Related Adhesive Protein (TRAP), which shows an increase in the number of osteoblasts and inhibition of osteoclast action. However, the treated orchiectomized groups did not obtain such expressive results. (4) Conclusion: The use of Biogran^®^ with PTH improved alveolar repair in rats. However, new researches with more efficient doses must be studied to collaborate effectively with the formation of a quality bone after the orchiectomy.

## 1. Introduction

Osteoporosis is a common disease in women over the age of 50, affecting up to a third of this population [1]. However, this is not a rare disease in men; it is estimated that men over the age of 50 lose up to 1% bone mineral density (BMD) per year, and one in eight older men developing osteoporosis during their lifetime [2,3,4]. The mechanisms related to bone loss and potential fracture risk in men, in general, are caused by decreased BMD, senility, smoking, excessive alcohol use, diabetes, decreased intestinal calcium absorption, and hypogonadism [2,3,5,6,7,8,9,10].

Several effective treatments have been found to control less severe osteoporosis, such as antiresorptive agents (denosumab, strontium ranelate or bisphosphonates) [3,11]. However, in cases of advanced osteoporosis, teriparatide (Forteo, Eli Lilly, Indianapolis, IN, USA), known as PTH (1-34), should be considered the first choice for treatment. Systemic PTH is the only osteoforming agent approved for the treatment of severe osteoporosis in men; it stimulates osteoblastic activity by increasing bone formation, increases trabecular and cortical thickness, and reduces fracture risk [3,11,12,13,14,15,16].

The topical action of PTH is being studied and has shown promising results in local injections into post-exodontic alveoli of rats, promoting increased bone filling and improved alveolar bone reconstruction [17]. Its topical use has been tested and proven to release teriparatide in concentrations sufficient to effectively influence bone cell function, suggesting potential efficacy of local PTH 1-34 availability [15].

Most of the clinical conditions found for the oral rehabilitation of patients are the need to install implants after immediate extraction or areas of low bone quantity and quality [18]. For this, there was a requirement for advances to prepare the recipient bed through autogenous, xenogenous or alloplastic grafts [19] that promote osseointegration [20,21]. Among the alloplastic materials, one of the options is Biogran^®^ (Biomet 3i, Palm Beach Gardens, FL, USA), which is composed of fully synthetic bioactive glass and does not cause risk of disease transmission; it is an osteoconductive material that stimulates the promotion of bone formation cells [22,23,24,25,26]. It has been certified to be easy to manipulate and to provide excellent hemostasis of the operative field [27]. In addition, the alloplastic biomaterial can be used as a graft in bone defects (alveoli) to promote osseointegration of titanium implants and the preservation of alveolar bone ridge height [18,20].

The improvement of biomaterials properties, through the incorporation of biomolecules that may evoke cellular responses that will be contributing to the repair process, is a challenge that has presented interesting results. Teriparatide that already presented important results through systemic administration has also presented an activation of the osteoblasts receptor and has become an attractive biomolecule that may be delivered in the repair site and may develop interesting responses activating bone formation [12,13,14,28,29]. Studies with topical application of teriparatide presented positive responses in bone formation [17]. Studies in the literature have evaluated the biological effect of biomaterials functionalized with biomolecules in the repair process; there are still doubts about their real clinical effectiveness. Lisboa-Filho et al. (2018) [24] concluded that Biogran^®^ loaded with raloxifene favored bone formation in calvarial defects. Deliberador et al. (2018) [30] concluded that the use of local PTH or alendronate added to particulate bone graft had no promising effect on bone acceleration and regeneration, but that local PTH use showed slight induction of bone formation with better results than local bisphosphonate treatment. Toker et al. (2012) [31] reported that the use of autogenous graft embedded in alendronate promoted increased bone formation and inhibition of osteoclastic activity in calvarial defects. Ozer et al. (2017) [32] presented results in which local administration of alendronate with autogenous graft helped to retain the graft in the defect and improve bone formation and osteoconductive properties of the graft in rabbit mandibular body defects. The functionalization of Biogran^®^ with PTH 1-34 is a modern study with no publications in the literature to date, which makes this work a novel report.

Thus, in view of the increasing population in dental offices, it is essential to check for medications that promote bone formation and enable the maintenance of the surgical bed, establishing a clinical protocol for the treatment of patients with osteoporosis [33,34,35]. This study has the advantage of an innovative approach, where we evaluated the use of a graft biomaterial loaded with an osteoforming medication, which was able to tell us the benefits that this union can promote in osteoporotic individuals.

In view of the above, the aim of this study was to evaluate topical PTH 1-34 functionalized to Biogran^®^ in the alveolar repair process after exodontia in rats with osteoporosis.

## 2. Materials and Methods

### 2.1. Experimental Groups

This study followed the Ethical Principles of Animal Experimentation and its execution was approved by the Ethics Committee on Animal Use (CEUA) of the Araçatuba Dental School with registration number (00199-2017) following the standards established by the ARRIVE Guidelines [36]. Ninety-six male rats (Rattus novergicus albinus, Wistar) weighing approximately 300 grams and aged approximately three months were divided into two groups according to the osteoporosis induction surgery: 48 rats undergoing sham surgery (SHAM) and 48 rats undergoing bilateral orchiectomy surgery (ORQ). The SHAM and ORQ groups were divided into three different subgroups: CLOT (no biomaterial was used in the post-exodontic alveolus, which was filled with blood clot only); BG (the alveolus was filled with Biogran^®^); BG-PTH (the alveolus was filled with Biogran^®^ associated with topical teriparatide). These animals were kept in cages at a temperature of 22 °C, on a balanced diet (NUVILAB, Curitiba, PR, Brazil) containing 1.4% Ca and 0.8% P and water ad libitum throughout the study.

### 2.2. Sample Calculation and Randomization

The sample size calculation for this study was determined from a power test carried out on the website http://www.openepi.com/SampleSize/SSMean.htm (OpenEpi, Version 3, open-source calculator; acceseed on 6 December 2021). This test was based on a study published in 2020 by Xu Lin et al. [37]: where data referring to the Micro-CT were used, in which the mean for group A = 2.37 and for group B = 1.52 and standard deviation for A = 0.29 and B = 0.14, with a significance level of 5% and power of 95% in a one-tailed hypothesis test; the sample size was smaller than that proposed in the present study.

All animals allocated to the facilities of the Araçatuba School of Dentistry were randomly assigned to their groups. Randomization was performed by an author (P.H.S.G.F) using Microsoft Office Excel software (Microsoft, Redmond, WA, USA). During sham surgery or orchiectomy the animals were randomly assigned without interference by the authors. The distribution of the ependorfs containing the biomaterial processed or not with PTH, 1-34 for insertion into the post-exodontic alveolus was randomly distributed to each animal, being only controlled so that there would be no failure in the distribution of the animals that would receive Biogran^®^ alone or Biogran^®^ + PTH. The insertion of the biomaterial into the cavity was always performed by the same surgeon (F.R.d.S.B), who was experienced in the technique. Only the right hemi-maxilla was used for all the analyses and the hemimaxillas were individually and randomly distributed between the Micro-CT/fluorochromes or immunohistochemistry analyses. The left hemi-maxilla was discarded because the removal of the left upper incisor was impossible when the right upper incisor was extracted.

### 2.3. Nano-Scale Reduction of PTH 1-34 and Biogran^®^

Mixing and homogenization of the samples was performed by sonochemistry, using either pure Biogran^®^ and Milli-Q^®^ water or Biogran^®^ + PTH 1-34 and ultrapure Milli-Q^®^ water to reduce particle size and obtain a homogeneous system. For ultrasonic processing, a Sonics VCX-750 model was used, 750 W power and 20 kHz frequency, with 5-minute pulses and 1-minute rest three times, totaling 15 min and variable amplitude set up to 40% of the equipment nominal amplitude (450 W/cm^2^). The samples were labeled as BG and BG-PTH and were subsequently oven dried at 60 °C and sterilized under UV radiation [38]. The relative mass concentration of PTH and Biogran^®^ was determined following standard described in 2018 by Tao et al. [39] and a dose of ≈2 μL of PTH 1-34 to 157 mm^3^ of Biogran^®^ was used.

### 2.4. Bilateral Orchiectomy

All rats were initially anesthetized with Coopazine (Xilazine-Coopers, Brazil, Ltd., Osasco, SP, Brazil) and Vetaset (ketamine hydrochloride, injectable, Fort Dodge, Animal Health, Ltd., Campinas, SP, Brazil) at the dosages recommended by the manufacturer. After anesthesia, antisepsis was performed with Polyvinyl pyrrolidone iodide (PVPI 10%, Riodeine Degermante, Rioquímica, São José do Rio Preto, SP, Brazil).

In the ORQ group the incisions were made in both scrotal sacs, with the testes being exposed. With the hemostatic forceps the spermatic funiculus was presented, with simultaneous individualization, lassoing and sectioning of the ductus deferens and the vascular pedicle. The testicles were removed, and the surgical wounds were sutured with polyglactin 910 4-0 thread (Ethicon, Johnson & Johnson, São José dos Campos, SP, Brazil). This procedure was validated in 2015 by Seifi et al. [40] after proof of a decrease in endogenous testosterone by Elisa test. The rats in the SHAM group underwent the same procedure, but only the surgical exposure of the testes was performed without removing them, in order to subject the animals in this group to the same surgical stress as the ORQ group [41]. Postoperatively, benzathine penicillin G for veterinary use was administered in a single intramuscular dose (0.2 mL/kg, Fort Dodge Animal Health Ltd., Campinas, SP, Brazil).

### 2.5. Tooth Extraction

**Figure 1:**
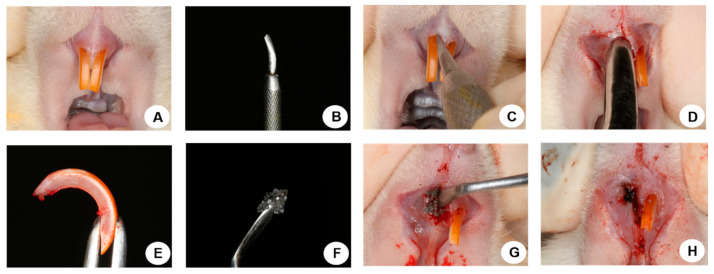
Extraction Surgery and Biomaterial Insertion. (**A**) Upper incisors of the animal; (**B**) Instrumental; (**C**,**D**) Dislocation and extraction of the upper right incisor; (**E**) Upper right incisor removed (extraction); (**F**) Biomaterial; (**G**) Insertion of the biomaterial in the alveolus; (**H**) Suture.

Thirty days after orchiectomy and sham surgery the animals were fasted for eight h prior to the surgical procedure and sedated with Coopazine (Xilazine-Coopers, Brazil, Ltd., Osasco, SP, Brazil) and Vetaset (ketamine hydrochloride, injectable, Fort Dodge, Animal Health, Ltd., Campinas, SP, Brazil) and received mepivacaine hydrochloride (0.3 mL/Kg, Scandicaine 2% with adrenaline 1:100,000, Septodont, Saint-Maur-des-Fossés, France) as local anesthesia. Antisepsis of the region was performed with polyvinyl pyrrolidone iodine (PVPI 10%, Riodeine Degermante, Rioquímica, São José do Rio Preto, SP, Brazil). With the help of instruments specially adapted in 1973 by Okamoto et al. [35], the dislocation was performed with subsequent exodontia of the right upper incisor. The SHAM and ORQ groups were subdivided into subgroups (CLOT, BG, and BG-PTH). For the CLOT subgroup, no biomaterial was used in the post-exodontic alveolus, only blood clot formation took place. The BG subgroup had the alveolus filled with Biogran^®^ and BG-PTH, filled with Biogran^®^ associated with topical PTH 1-34. The volume of biomaterial used to fill the empty alveoli in the BG and BG-PTH subgroups occupied the cervical and middle thirds. The gingival mucosa was sutured with polyglactin 910 thread (Vicryl 4.0-Jhonson & Jhonson, New Brunswick, NJ, USA) (Figure 1). The pentabiotic was administered (0.2 mL/kg, Fort Dodge Animal Health Ltd., Campinas, SP, Brazil) in a single intramuscular dose in the immediate postoperative period. The animals were maintained with ground feed (NUVILAB, Curitiba, PR, Brazil) until the end of the experiment.

### 2.6. Application of Fluorochromes

At 14 days after exodontia of the right upper incisor, 20 mg/kg [34] of Calcein was administered intramuscularly. After 28 days (42 days after exodontia), Alizarin Red fluorochrome was administered intramuscularly in the amount of 20 mg/kg [42].

### 2.7. Collection of Materials

After 60 days from surgery, the rats were euthanized. After euthanasia, the right maxillae were removed and reduced for computerized microtomography, laser confocal microscopy, and immunolabeling analyses.

### 2.8. Micro-CT Analysis

For the Micro-CT analysis after the animals were euthanized, the right maxillae were removed, fixed in 10% formalin (Dinâmica Odonto-Hospitalar Ltd., Catanduva, SP, Brazil) for 48 h, washed for 24 h, reduced to the area of alveolar repair, and stored in 70% alcohol. These parts were first subjected to X-ray beam scanning analysis in a digital computerized microtomography system, scanned by SkyScan microtomograph (SkyScan 1272 Bruker Micro CT, Aartselaar, Antwerp, Belgium, 2003) using 12 µm thick sections (70 Kv and 142 μA), with a 0.5 mm aluminum filter and a rotation step of 0.4° at a time of 37 min. The images obtained by projection of X-rays on the samples were stored and reconstructed determining the area of interest by NRecon software (SkyScan, 2011; Version 1.6.6.0, Kontich, Antwerp, Belgium). In the Data Viewer software (SkyScan, Version 1.4.4 64-bit, Kontich, Antwerp, Belgium), the images were reconstructed to fit the standard positioning for all samples and can be observed in the transaxial plane. Then, the CTAnalyser-CTAn software (2003-11SkyScan, 2012 Bruker Micro CT Version 1.12.4.0, Kontich, Antwerp, Belgium) that measures the image according to gray scale (threshold) was used. The threshold used was 140-55 shades of gray, which makes it possible to obtain the volume of bone formed in the alveoli under repair [34].

The images obtained from the computed microtomography were used to characterize the parameters of volume (BV), percentage of bone volume (BV/TV), trabecular thickness (Tb.Th), number of trabeculae (Tb.N), separation of trabeculae (Tb.Sp) and percentage of total porosity (Po(tot)), using the CTanalyser software [43]. Finally, with the CTvox software (SkyScan, version 2.7, Kontich, Antwerp, Belgium) the 3D reconstruction was performed.

### 2.9. Confocal Laser Microscopy

After Micro-CT, the same parts were submitted to increasing alcohol dehydration (70%, 90% and 100%), changing the solution every five days with incubation in an orbital shaker (KLine CT-150^®^, Cientec-Laboratory Equipment, Piracicaba, SP, Brazil) daily for four hours.

After dehydration, the specimens were infiltrated in a solution of acetone and slow methyl methacrylate (PMMAL) (Clássico, Artigos Odontológicos Clássico, São Paulo, SP, Brazil) in a 1:1 ratio. Then, they received 3 baths of PMMA; in the last bath, the catalyst benzoyl peroxide 1% (Riedel-De Haën AG, Seelze-Hannover, Lower Saxony, Germany) was added. The last bath (PMMAL and catalyst) was carried out with the parts placed in glass jars with lids, which were kept in an oven at 37 °C for 5 days until the resin was polymerized.

After polymerization, the vials were reduced parallel to the long axis of the hemimaxilla with a “Maxcut” drill mounted on a Kota benchtop motor (Strong 210, São Paulo, SP, Brazil). Next, progressive grinding was performed in an automatic polisher (ECOMET 250 PRO/AUTOMET 250, Buehler, Lake Bluff, IL, USA) up to a thickness of 80 μm. A digital caliper was used for measurement (Mitutoyo, Pompeia, SP, Brazil). The obtained sections were mounted on histological slides.

The slides were scanned with a confocal laser scanning microscope (Leica CTR 4000 CS SPE, Leica Microsystems, Heidelberg, Baden-Württemberg, Germany) through the longitudinal hemimaxillary sections of the alveolar bone adjacent to the apical third of the maxillary central incisor using a 10× objective (original magnification 100).

Fluorochromes are chemical compounds that have the property of binding to calcium at the time of precipitation in the bone matrix. Thus, the extent of the fluorochrome labeling represents the amount of calcium precipitation in the matrix, which allows for measuring the amount of bone formed. Another aspect to be considered is the period in which the fluorochromes were injected; the calcein injected earlier has the bone marked (calcium precipitation) with the green fluorochrome and, in this case, represents an older bone. The r last fluorochrome injected was alizarin, with bone marked (calcium precipitation) by red; it represents new bone, meaning the active mineralization surface is difference between old bone (green) and new bone (red) [44]. Therefore, it can be said that the different colors represent the different periods of bone formation.

Thus, calcein and alizarin red fluorochrome images separately (old bone/new bone, respectively) and by overlay were used to evaluate the mineral apposition rate (MAR), the area of neoformed bone (NBA) and alveolar bone histometry and dynamics.

The images saved in TIFF format were transported to ImageJ software (Image Processing and Analysis Software, Bethesda, MD, USA). With the straight tool, the MAR was found through measurements that extended from the calcein margin toward the alizarin margin; the value obtained was divided by 28, which represents the interval of days between the injections of the analyzed fluorochromes [45]. To measure the area of neoformed bone, the overlay of the images and the “measura” tool (μm^2^) were used.

### 2.10. Immunolabeling Analysis

After euthanasia, the removed specimens were fixed in 10% formaldehyde solution (Reagentes Analiticos, Dinâmica Odonto-Hospitalar Ltd., Catanduva, SP, Brazil) for 48 h, washed in tap water for 24 h, and decalcified in 20% EDTA for 5 weeks. Then, dehydration was performed using a sequence of alcohols. The pieces were diaphanized in xylene for subsequent paraffin embedding to obtain sections 6 μm thick, and then were mounted on slides.

The reaction was performed by the indirect immunoperoxidase method with an amplifier, the endogenous peroxidase activity was inhibited with hydrogen peroxide. Then, the slides went through the antigenic recovery step with citrate phosphate buffer (pH 6.0) kept in warm humidity for 40 min; the nonspecific blocking of the reaction was performed with bovine albumin (Sigma). The primary antibodies (Dilution 1:100) used were against Wnt, Beta catenin (β-catenin), osteocalcin (OC), osteoprotegerin (OPG), RANKL and TRAP, polyclonal antibodies produced in goats (Santa Cruz Biotechnology). With secondary antibody (dilution 1:200) biotinylated anti-goat antibody produced in rabbit (Pierce Biotechnology), the amplifier was Streptavidin and Biotin (Dako) and diaminobenzidine (Dako) as chromogen. At the end of the reaction, the slides were counterstained with Meyer’s hematoxylin.

For each of the antibodies used, protein expression was evaluated semi-quantitatively (ordinal qualitative analysis) by assigning different scores according to the number of immunolabeled cells in the alveolar repair process. The analysis was performed under an optical microscope (LeicaR DMLB, Heerbrugg, Sankt Gallen, Switzerland), using scores that represented: light staining (1), moderate staining (2) and intense staining (3), the staining with diaminobenzidine being considered as positive, taking care to maintain negative controls to evaluate the specificity of the antibodies [46,47]. These scores were established according to the labeling: mild represented about 25% of the immunolabeling area; moderate represented about 50% of the area; intense represented about 75% of the area [34].

A single examiner (P.B.F) performed the laboratory processing of the immunohistochemistry slides and another calibrated examiner (R.O) performed all the immunohistochemical analyses to validate the results. The second examiner (R.O) was not aware of the groups or proteins (blinding). Intra-examiner reliability was analyzed with 24-h duplicate evaluations, in which Kappa test (K > 0.80) was performed on all histological slides.

Immunolabeling was performed to characterize the different stages of development and maturation of osteoblasts during the alveolar repair process. Thus, positive markings for Wnt and Beta Catenin relate to the later stages of bone tissue formation, being evidenced the differentiation, activation and recruitment of osteoblasts [48], and the OC protein, which is a protein of the extracellular matrix that is expressed in later periods, when calcium is precipitated in the bone tissue, in a more mature stage [34]. Positive markings for OPG and RANKL signal a balance in the bone remodeling process with positive marking for osteoblasts and osteoclasts [48]. TRAP characterizes osteoclastic activity in the alveolus under repair [34]. The aim of immunostaining was to evaluate whether treatment with topical PTH 1-34 functionalized to Biogran^®^ had contributed to accelerated maturation of osteoblasts that actively participate in bone repair responses.

### 2.11. Statistical Analysis

The GraphPad Prism 7.03 (GraphPad Software, San Diego, CA, USA) software was used for statistical analyses. To evaluate the parameters of Micro-CT, MAR and NBA, two-way ANOVA was used, considering two factors: systemic condition (SHAM vs. ORQ), using the Holm–Sidak post-test, and the biomaterial factor (CLOT vs. BG vs. BG-PTH), using Tukey’s post-test to recognize differences between groups. For histometry and alveolar bone dynamics, the two-way ANOVA test and the Tukey post-test were used, with no statistical difference between the groups; therefore, the one-way ANOVA test was applied to compare calcein and alizarin fluorochromes between all groups, with Tukey’s post-test. The significance level was set at *p* < 0.05.

## 3. Results

### 3.1. Micro-CT

**Figure 2:**
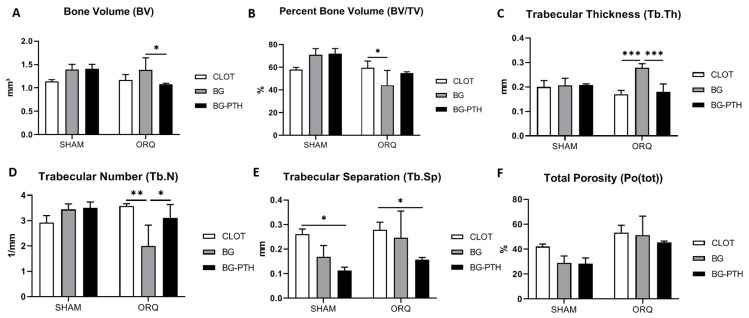
Micro-CT analyses. (**A**) Bone volume (BV). (**B**) Percentage of bone volume (BV/TV). (**C**) Trabecular thickness (Tb.Th). (**D**) Trabecular number (Tb.N). (**E**) Trabecular separation (Tb.Sp). (**F**) Total Porosity (Po(tot)). Asterisks (*,**,***) denote intragroup statistical difference (*p* < 0.05). The intergroup difference can be seen in Table 1.

There was a statistically significant difference for bone volume (BV) (*p* > 0.05, two-way ANOVA). For the systemic condition factor, SHAM vs. ORQ showed a statistical difference for the BG-PTH biomaterial (*p* = 0.0261, Holm–Sidak) (Table 1). In the biomaterial factor, in the ORQ group, there was a difference between BG vs. BG-PTH (*p* = 0.0339, Tukey). The numerical values obtained for the groups were similar; however, the SHAM BG-PTH group (BV = 1.366 mm^3^) had greater bone volume when compared to the other groups (Figure 2A and Table 2).

In the percentage of bone volume (BV/TV), for the systemic condition factor, SHAM vs. ORQ showed a statistically significant result for BG (*p* = 0.0010, Holm–Sidak) and BG-PTH (*p* = 0.0257, Holm–Sidak) (Table 1). For the biomaterial factor, in the ORQ group there was a statistically significant between CLOT vs. BG (*p* = 0.0388, Tukey). The SHAM BG-PTH group obtained the highest numerical value of percentage bone volume (BV/TV = 69.575%) (Figure 2B and Table 2).

For the thickness of the trabeculae (Tb.Th), the systemic condition factor, SHAM vs. ORQ showed a statistically significant difference for the BG biomaterial (*p* = 0.0067, Holm–Sidak) (Table 1). For the biomaterial factor, in the ORQ group, there was statistically significant difference between CLOT vs. BG (*p* = 0.0002, Tukey); BG vs. BG-PTH (*p* = 0.0005, Tukey). The group with the greatest thickness between trabeculae was ORQ BG (Tb.Th = 0.266 mm) (Figure 2C and Table 2).

For number of trabeculae (Tb.N), the systemic condition factor, SHAM vs. ORQ showed a statistically significant difference for BG (*p* = 0.0047, Holm–Sidak) (Table 1). For the biomaterial factor, in the ORQ group, there was a statistically significant difference between CLOT vs. BG (*p* = 0.0022, Tukey) and BG vs. BG-PTH (*p* = 0.0222, Tukey). The numerical values obtained for the groups were similar; however, the SHAM BG-PTH (Tb.N = 3.365 L/mm) and ORQ CLOT (Tb.N = 3.345 L/mm) groups obtained a greater number of trabeculae when compared to the other groups (Figure 2D and Table 2).

In the separation between the trabeculae, for the systemic condition factor, SHAM vs. ORQ, no statistically significant result was observed in the assessments of biomaterials (Table 1). For the biomaterial factor, in the SHAM group, there was a statistically significant result between CLOT vs. BG-PTH (*p* = 0.0102, Tukey). In the ORQ group, there was a statistically significant result between CLOT vs. BG-PTH (*p* = 0.0311, Tukey). The SHAM BG-PTH group was the one with the lowest value for trabecular separation (Tb.Sp = 0.123 mm) (Figure 2E and Table 2).

For total porosity (Po(tot)), the systemic condition factor, SHAM vs. ORQ, differences were observed in the biomaterial BG (*p* = 0.0094, Holm–Sidak) and BG-PTH (*p* = 0.0458, Holm–Sidak) (Table 1). For the biomaterial factor, no statistically significant differences were observed in the comparisons made. The ORQ group presented bone with greater porosity when compared to the groups of healthy animals and the SHAM BG-PTH group presented the smallest porosity (Potot = 30.423%) (Figure 2F and Table 2).

### 3.2. Confocal Laser Microscopy

**Figure 3:**
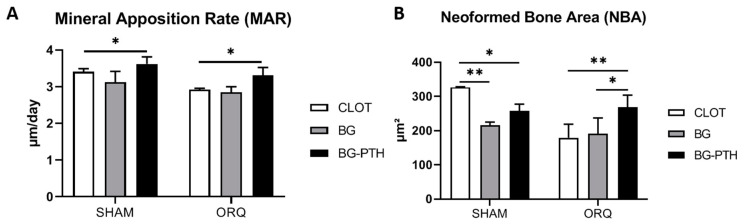
Fluorochrome analysis. (**A**) Mineral apposition rate (MAR). (**B**) Bone area formed (NBA). Asterisks (*,**) denote intragroup statistical difference (*p* < 0.05). The intergroup difference can be seen in Table 1.

For the mineral apposition rate (MAR), the systemic condition factor, SHAM vs. ORQ, statistically significant difference in the CLOT biomaterial were observed (*p* = 0.0230, Holm–Sidak) (Table 1). For the biomaterial factor, statistically significant differences were observed in the SHAM group, when comparing BG vs. BG-PTH (*p* = 0.0155, Tukey). In the ORQ group, a statistically significant difference was observed when comparing BG vs. BG-PTH (*p* = 0.0258, Tukey). The group with the highest MAR was SHAM BG-PTH (3.623 µm/day) (Figure 3A and Table 2).

Regarding the neoformed bone area, for the systemic condition factor, SHAM vs. ORQ, statistically significant differences were observed in the CLOT biomaterial (*p* = 0.0002, Holm–Sidak) (Table 1). For the biomaterial factor, differences were observed in the SHAM group, when comparing CLOT vs. BG (*p* = 0.0020, Tukey) and when comparing CLOT vs. BG-PTH (*p* = 0.0402, Tukey). In the ORQ group, there was a statistically significant difference in comparison between CLOT vs. BG-PTH (*p* = 0.0096, Tukey) and when compared BG vs. BG-PTH (*p* = 0.0214, Tukey). It was possible to notice in the SHAM CLOT group a balance between the amount of new and old bone that was formed after the application of fluochromes (Figure 3B and Table 2).

**Figure 4:**
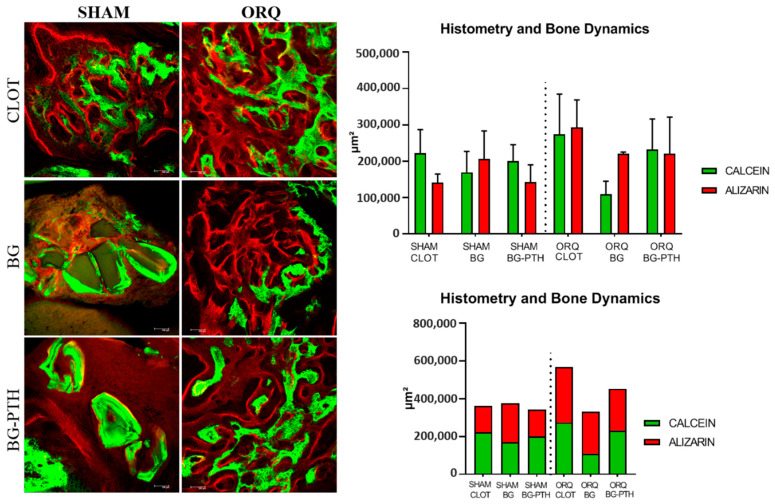
Confocal microscopy analysis the fluorescent areas in the calcium matrix by overlap of calcein (green) and alizarin (red). Graphic representation of the bone dynamics of the experimental groups: SHAM and ORQ (CLOT, BG and BG-PTH). The statistical difference can be seen in Table 3.

As for histometry and bone dynamics, the groups that had greater formation of new bone (alizarin) were SHAM BG (242.846 µm^2^) and ORQ BG (221.026 µm^2^) when compared to the formation of old bone (calcein), which was proportionally less. When comparing the intergroup and intragroup relationship, there was a statistically significant difference, as can be seen in Table 3 (Figure 4 and Table 3).

### 3.3. Immunolabeling Analysis

**Figure 5:**
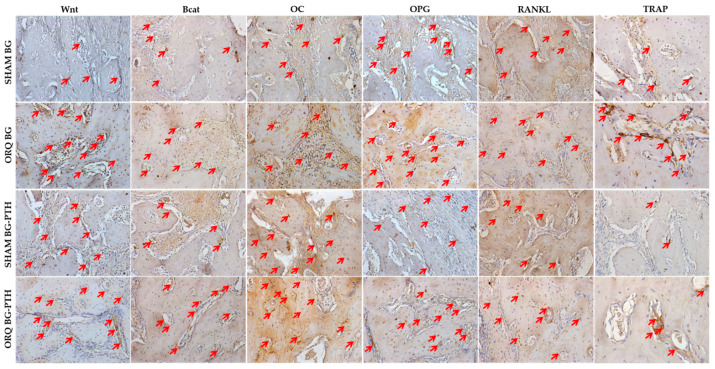
Immunohistochemical analysis of SHAM and ORQ groups (BG and BG-PTH) through protein labeling: Wnt, Bcat, OC, OPG, RANKL and TRAP. Labeling indicated by red arrows, (Original, 20×).

The representation of the scores observed in all groups are presented in Table 4. Scores representing arrows can be seen in Figure 5 for all SHAM and ORQ groups (BG and BG-PTH) and all studied proteins (Figure 5). Brief description of the biological activity of six proteins can be seen in Table 5.

The expression of the Wnt protein present in osteoblasts during the alveolar repair process was slightly expressed (1) in the SHAM CLOT, ORQ CLOT and SHAM BG groups, the ORQ BG, SHAM BG-PTH and ORQ BG-PTH groups were moderately immunolabeling (2).

Beta catenin protein present during the bone repair process, for the SHAM CLOT, ORQ CLOT SHAM BG and SHAM BG-PTH groups, presented with light labeling (1), and moderate labeling (2), was observed in the ORQ groups BG and ORQ BG-PTH.

For immunolabeling of OC protein, a non-collagenous and more abundant protein in the extracellular matrix related to late periods of osteoblast differentiation, moderate labeling (2) was observed for SHAM CLOT, ORQ CLOT, SHAM BG and ORQ BG and intense labeling (3) for SHAM BG-PTH and ORQ BG-PTH.

Regarding the bone remodeling process, the OPG protein showed mild labeling (1) for SHAM CLOT, ORQ CLOT and SHAM BG-PTH, moderate (2) for SHAM BG, ORQ BG-PTH and intense (3) for ORQ BG. For RANKL protein, SHAM CLOT, ORQ CLOT, SHAM BG, SHAM BG-PTH and ORQ BG-PTH groups showed moderate (2) and intense (3) labeling for ORQ BG.

On osteoclastic activity, TRAP protein showed mild (1) labeling for SHAM CLOT, ORQ CLOT SHAM BG and SHAM BG-PTH and moderate (2) for ORQ BG-PTH and intense (3) for ORQ BG.

## 4. Discussion

The orchiectomy performed in animals induces osteopenia [40], because there is a decrease in the production of endogenous testosterone, which causes a decrease in the aromatization of testosterone into estradiol, causing the impairment of bone metabolism and, in consequence, bone resorption predominantes on bone formation [49,50,51]. This factor predisposes osteoporosis conditions, which affects 1 in every 8 men over the age of 50 [2,3,4], because several causes, such as hypogonadism, comorbidities, diabetes, and other factors can lead to decreased production of the male hormone and consequently the development of this disease [3,6,9].

Osteoporosis is a disease that affects the bone structure of the body, reducing bone quality and compromising its strength, thus increasing the risk of osteoporotic fractures [52]. This disease is still a matter of discussion in the literature regarding bone remodeling and alveolar reconstruction, as it is associated with a delay in the repair process of the dental cavity [34,48]. An important issue is to obtain more answers on how to produce a good quality mineralized tissue to support the mechanical forces arising from the rehabilitation of a prosthetic implant in osteopenic patients [28,53,54].

Currently, therapies against osteoporosis are already well established in the literature and teriparatide emerges with a new perspective, because it promotes the induction of cells responsible for the formation of new bone [55]. As it is an osteoforming agent, it will act directly on the osteoblastic cells, leading to an increase in BMD, making the bone more resistant to non-traumatic fractures [12,13,14,15,16].

In this study, Biogran (bioactive glass) was the biomaterial chosen to be loaded with teriparatide, considering the osteoconductive capacity, promoting hemostasis of the grafted area and being free from contamination [22,23,24,25,56]. Gomes-Ferreira et al. (2019) [26] validated the sonication time of Biogran^®^ at 15 min. The authors reported that this functionalization time caused the particles of the biomaterial to become smaller and more homogeneous, thus being able to promote peri-implant bone reconstruction and induce bone formation in better quality and quantity [26]. Lisboa-Filho et al. (2018) [24], concluded that the functionalization of Biogran^®^ with raloxifene favored quality bone formation in rat calvarial defect.

However, we should take into consideration that when there is no systemic alteration, the clot is the most favorable medium for repair [57,58], since the use of biomaterials can hinder the reparational response of the tissue. However, in cases where there is loss of buccal bone wall and implant installation is the procedure of choice for rehabilitative treatment, the maintenance of this bone is essential for a favorable prognosis, which is the main justification for the use of biomaterials and anabolic drugs. Therefore, we must choose drugs that have properties that most closely resemble the biological behavior of blood clots, thus justifying the use of Biogran^®^ augmentation with teriparatide.

De Oliveira et al. (2019) [59] observed that the teriparatide intermitent treatment in orchiectomized rats promoted an increase of bone volume percentage, as well as the improvement of trabeculae thickness, evidencing the positive effect on the formation of healthy bone. When evaluating whether the reparative alveolus filled with Biogran^®^ functionalized or not with PTH, 1-34 it was seen that there was also quality bone formation. The SHAM BG and SHAM BG-PTH groups obtained higher values for volume and percent bone volume (BV and BV/TV), less trabecular thickness (Tb.Th), less separation between trabeculae (Tb.Sp), a larger number of trabeculae (Tb.N) and less total porosity (Po (tot)). Orchiectomized animals showed less percent bone volume and separation between trabeculae and greater porosity of trabeculae, because they were induced to osteopenia; however, the use of topical Biogran^®^ + PTH helped discretely in the reconstruction of the alveolus, showing similar results to the SHAM CLOT group, where even for some parameters the treated ORQ group was superior to untreated orchiectomized rats (higher Tb.N and less Po.tot).

In laser confocal microscopy, for the mineral apposition rate (MAR), in which we observed the calcium precipitation on bone surface, we observed an intragroup relationship for SHAM BG-PTH and ORQ BG-PTH in which the values presented increased, demonstrating a clear improvement in bone repair after biomaterial insertion with local PTH. This can be compared with the systemic effect of teriparatide described in the study by De Oliveira Puttini et al. 2019 [29], in which the osteopenic group treated with systemic PTH also showed excellent results. For AON, the group that obtained the highest result was SHAM CLOT, with greater balance between new (red) and old (green) bone formation; the SHAM BG-PTH and ORQ BG-PTH groups were similar to it. For bone dynamics, the groups that showed the greatest formation of new bone (alizarin) were the groups with only the grafted biomaterial. The groups with the addition of PTH 1-34 to Biogran^®^ had no exponential improvement in this condition, since the addition of substances in inadequate amounts may disturb the bone formation process in some way. The same negative result was seen in the healthy untreated group, where old bone (green) outweighed new bone formation (red).

Bone proteins involved in alveolar repair responses (Wnt, Beta Catenin and OC), indicating higher labeling after Biogran^®^ functionalization with teriparatide, proving that osteoblastic activity was present. Regarding osteoclastic activity, OPG and RANKL proteins showed balanced expression in the BG-PTH subgroup for SHAM and ORQ. In addition, TRAP showed mild labeling for the treated groups, showing a local anabolic effect of teriparatide in opposition to osteoclast resorption activity. Studies of de Oliveira et al. and Oliveira Puttini et al. showed that the use of systemic teriparatide after exodontia in orchiectomized rats led to increased expression for Wnt and osteocalcin proteins [59], a balance between bone formation and resorption for OPG and RANKL proteins [29] and lower labeling for TRAP protein [59], indicating increased repair tissue formation and less alveolar bone resorption. Therefore, from the results presented, it can be inferred that the use of Biogran^®^ promoted slight improvement in rat alveolar repair and that, with the addition of PTH 1-34 topic, this condition tends to improve, resembling bone repair by clot formation.

Biogran^®^ (bioactive glass) is an excellent osteoinductive and osteoconductive biomaterial, which is used in the reconstruction of bone defects caused by periodontal disease [22,25]. When Biogran^®^ was loaded with teriparatide it resulted in slowing down the function of osteoclast cells. Although these medications are excellent for bone formation and maintenance, we believe that there may have been interferences and limitations, because when Biogran^®^ was loaded with teriparatide this functionalization may have limited its osteoconductive effect, leading to less expressive results. Moreover, because PTH is released slowly, it may have mimicked its intermittent effect, where this form of administration led to its anabolic effect. In a condition where there is a greater challenge in bone repair, as well as in systemic alteration, it may have triggered a better response caused by the functionalized biomaterial alone.

Thus, some limitations that can be highlighted in the present study are related to the performance of the graft technique of the biomaterial in the alveolus, since clot formation prevented the insertion of the biomaterial up to the apex and only the region of the middle third to the cervical of the alveolus was evaluated. Still, we believe that the chosen dose of parathormone may have been the major limiting factor for this study, otherwise, it is important to note that positive effects were observed during the alveolar repair process in orchiectomized animals.

Studies using more different concentrations should be conducted by our group for biological validation with other concentrations of teriparatide, so that osteopenia can be reversed with a more effective dose-dependent effect, since no positive effects were observed for some parameters. In addition, we should carry out tests with grafts associated with PTH for maxillary sinus lift or even filling of “gaps” during implant installation, since these techniques are quite consolidated in the literature; the functionalization of the biomaterial with parathormone is innovative and progress is still needed.

## 5. Conclusions

In view of the data reported in the present article, it can be concluded that the use of Biogran^®^ with topical PTH, 1-34 promoted improvement and maintenance of alveolar volume after extraction of the rat upper incisor. However, new studies using even more efficient concentrations of teriparatide should be carried out to effectively contribute to quality bone formation in osteopenic individuals.

## Figures and Tables

**Table 1:** Representation intergroup of the statistical difference between SHAM vs. ORQ (CLOT, BG and BG-PTH) for bone structure (BV, BV.TV, Tb.Th, Tb.N, Tb.Sp and Po(tot)) and bone dynamics (MAR and NBA), indicate significance at *p* < 0.05.

	Statistical Difference	*p* Value
Bone Struture		
BV (mm^3^)	SHAM BG-PTH vs. ORQ BG-PTH	0.0261
BV.TV (%)	SHAM BG vs. ORQ BG	0.0010
	SHAM BG-PTH vs. ORQ BG-PTH	0.0257
Tb.Th (mm)	SHAM BG vs. ORQ BG	0.0067
Tb.N (1/mm)	SHAM BG vs. ORQ BG	0.0047
Potot (%)	SHAM BG vs. ORQ BG	0.0094
	SHAM BG-PTH vs. ORQ BG-PTH	0.0458
Bone Dynamics		
MAR (µm/day)	SHAM CLOT vs. ORQ CLOT	0.0230
NBA (µm^2^)	SHAM CLOT vs. ORQ CLOT	0.0002

**Table 2:** Representation intragruop of the statistically significant difference between SHAM vs. SHAM (CLOT, BG and BG-PTH) and ORQ vs. ORQ (CLOT, BG and BG-PTH) for bone structure (BV, BV.TV, Tb.Th, Tb.N, Tb.Sp and Po(tot)) and bone dynamics (MAR and NBA), indicate significance at *p* < 0.05.

	Statistical Difference	*p* Value
Bone Struture		
BV (mm^3^)	ORQ BG vs. ORQ BG-PTH	0.0339
BV.TV (%)	ORQ CLOT vs. ORQ BG	0.0388
Tb.Th (mm)	ORQ CLOT vs. ORQ BG	0.0002
	ORQ BG vs. ORQ BG-PTH	0.0005
Tb.N (1/mm)	ORQ CLOT vs. ORQ BG	0.0022
	ORQ BG vs. ORQ BG-PTH	0.0222
Tb.Sp (mm)	SHAM CLOT vs. SHAM BG-PTH	0.0102
	ORQ CLOT vs. ORQ BG-PTH	0.0311
Bone Dynamics		
MAR (µm/day)	SHAM BG vs. SHAM BG-PTH	0.0155
	ORQ BG vs. ORQ BG-PTH	0.0258
NBA (µm^2^)	SHAM CLOT vs. SHAM BG	0.002
	SHAM CLOT vs. SHAM BG-PTH	0.0402
	ORQ CLOT vs. ORQ BG	0.0096
	ORQ CLOT vs. ORQ BG-PTH	0.0214

**Table 3:** Representation of bone dynamics: calcein vs. alizarin; calcein vs. calcein and alizarin vs. alizarin to represent the statistical difference between all groups SHAM vs. ORQ (CLOT, BG and BG-PTH), indicate significance at *p* < 0.05.

	Statistical Difference	*p* Value
Calcein vs. Alizarin	SHAM CLOT vs. ORQ CLOT	0.0008
	ORQ CLOT vs. ORQ BG-PTH	0.0026
	SHAM BG-PTH vs. ORQ CLOT	0.0016
	ORQ CLOT v. ORQ BG	0.0002
	SHAM BG vs. ORQ CLOT	0.026
	SHAM BG-PTH vs. ORQ CLOT	0.0361
Calcein vs. Calcein	ORQ CLOT vs. ORQ BG	0.0001
	SHAM BG vs. ORQ CLOT	0.0154
	SHAM BG-PTH vs. ORQ CLOT	0.0216
	SHAM CLOT vs. ORQ CLOT	0.0408
Alizarin vs. Alizarin	SHAM CLOT vs. ORQ CLOT	0.0014
	ORQ CLOT vs. ORQ BG-PTH	0.0045
	SHAM BG-PTH vs. ORQ CLOT	0.0027

**Table 4:** Scores observed in the marking of Wnt, β-Catenin, osteocalcin, osteoprotegerin, RANKL and TRAP for all SHAM and ORQ groups (CLOT, BG and BG-PTH).

	Wnt	β-Catenin	Osteocalcin	Osteoprotegerin	RANKL	TRAP
SHAM CLOT	1	1	2	1	2	1
ORQ CLOT	1	1	2	1	2	1
SHAM BG	1	1	2	2	2	1
ORQ BG	2	2	2	3	3	3
SHAM BG-PTH	2	1	3	1	2	1
ORQ BG-PTH	2	2	3	2	2	2

**Table 5:** Brief description of proteins Wnt, β-Catenin, osteocalcin, osteoprotegerin, RANKL and TRAP.

Proteins	Description
Wnt	Different stages of osteoblasts (differentiation, activation and recruitment)
β-Catenin	Different stages of osteoblasts (differentiation, activation and recruitment)
Osteocalcin	Bone maturation regulation process
Osteoprotegerin	Process and regulation of bone formation and resorption
RANKL	Process and regulation of bone formation and resorption
TRAP	Osteoclastic activity

## Abbreviations

Bcat	Beta Catenin
BG	Alveolar Defect with Biogran^®^
BG-PTH	Alveolar Defect with Topical Biogran^®^+PTH 1-34
Biogran^®^	Bioactive Glass-Based Biomaterial
BMD	Bone Mineral Density
BV	Bone Volume
BV/TV	Bone Volume Percentage
CEUA	Animal Use Ethics Committee
CLOT	Alveolar Defect without Biomaterial
CTan	CT Analyzer
EDTA	Ethylenediamine Tetra-Acetic Acid
ELISA	Enzyme Immunoadsorption Assay
FAPESP	State of São Paulo Research Support Foundation
FOA	Araçatuba Dental School
MAR	Daily Mineral Apposition
Micro-CT	Computerized Microtomography
NBA	Neoformed Bone Area
OC	Osteocalcin
OPG	Osteoprotegerin
ORQ	Orchiectomy Surgery
Ph	Hydrogen potential
PMMAL	Slow Methyl Methacrylate Acetone Solution
Po(tot)	Total Porosity
PTH, 1-34	Parathyroid Hormone
PVPI	Povidone Iodine
RANKL	Nuclear Factor Kappa B Activator Receptor Ligand
SHAM	Sham Surgery
Tb.N	Number of Trabeculae
Tb.Sp	Separation between Trabeculae
Tb.Th	Trabecular Thickness
TIFF	Tagged Image File Format
TRAP	Thrombospondin-Related Adhesive Protein
UNESP	São Paulo State University “Júlio de Mesquita Filho”
WNT	Wnt route
